# *ABCA1* 69C>T Polymorphism and the Risk of Type 2 Diabetes Mellitus: A Systematic Review and Updated Meta-Analysis

**DOI:** 10.3389/fendo.2021.639524

**Published:** 2021-04-23

**Authors:** Ha Young Yoon, Min Hye Lee, Yubin Song, Jeong Yee, Gonjin Song, Hye Sun Gwak

**Affiliations:** College of Pharmacy and Graduate School of Pharmaceutical Sciences, Ewha Womans University, Seoul, South Korea

**Keywords:** *ABCA1* 69 C>T, meta-analysis, systematic review, polymorphism, type 2 diabetes mellitus

## Abstract

**Background:**

The ATP-binding cassette transporter A1 *(ABCA1)* is likely associated with the risk of type 2 diabetes mellitus (T2DM) *via* β cell function modification, but the evidence on the association remains unclear. This study aimed to investigate the relationship between the *ABCA1* 69C>T polymorphism and the risk of T2DM through a systematic review and meta-analysis.

**Materials and Methods:**

The PubMed, Web of Science, and Embase databases were searched for qualified studies published until August 2020. Studies that included the association between the *ABCA1* 69C>T polymorphism and the risk of T2DM were reviewed. The odds ratios (ORs) and 95% confidence intervals (CIs) were evaluated.

**Results:**

We analyzed data from a total of 10 studies involving 17,742 patients. We found that the CC or CT genotype was associated with increased risk of T2DM than the TT genotype (OR, 1.41; 95% CI, 1.02-1.93). In the Asian population, the C allele carriers had a higher risk of T2DM than those with the TT genotype; the ORs of the CC and CT genotypes were 1.80 (95% CI, 1.21-2.68) and 1.61 (95% CI, and 1.29-2.01), respectively.

**Conclusions:**

This meta-analysis confirmed that the *ABCA1* 69C>T genotype showed a decrease risk of T2DM compared to the CC or CT genotypes.

## Introduction

Diabetes is a major global health issue estimated to have affected approximately 463 million people in 2019, with this number predicted to reach 700 million by 2045 according to the International Diabetes Federation (1). In addition, the annual cost of diabetes care is USD 760 billion (2). Diabetes is a serious, chronic endocrine disease that occurs when the blood glucose level is elevated due to insufficient insulin secretion and low sensitivity of target organs or cells to insulin (3–5). Diabetes is also associated with several comorbidities such as neuropathy and angiopathy, which have become leading causes of mortality and morbidity worldwide (1, 6).

Diabetes is classified into 2 types, and type 2 diabetes mellitus (T2DM) is the most prevalent. The development of T2DM is complex and involves a combination of several genetic and environmental factors (7–9). Several genes contribute to the overall susceptibility to T2DM by influencing the baseline glucose tolerance level (10). Genetic linkage analysis and association studies have identified several candidate genes contributing to T2DM.

The ATP-binding cassette transporter A1 (*ABCA1*) is considered an important gene that can modify β cell function, although its primary function is associated with cholesterol metabolism. ABCA1 contributes to the reverse transportation of cholesterol from peripheral tissues to the liver *via* high-density lipoprotein-cholesterol (HDL-C). Polymorphism in the *ABCA1* gene is reportedly related to HDL-C deficiency, which leads to coronary heart disease or coronary artery disease (11–13). *ABCA1* may also be crucial to maintaining β cell cholesterol homeostasis and function (14, 15). Because cholesterol is an important factor for membrane organization and survival of β cells, cholesterol accumulation in β cells impairs glucose metabolism and reduces insulin secretion, resulting in the development of T2DM (16). A study showed that β cell–specific *Abca1* knockout mice had significantly higher fasting blood glucose levels than their littermate controls because of a defect in the first-phase glucose-stimulated insulin release (17). Studies have shown that several common variants of *ABCA1* gene are also associated with the development of T2DM in humans (18). However, evidence regarding the association between *ABCA1* gene polymorphisms and the risk of T2DM remains unclear. Therefore, we aimed to investigate the relationship between the extensively studied *ABCA1* 69C>T polymorphism and the risk of T2DM through a systematic review and meta-analysis.

## Methods and Materials

### Literature Search Strategy

Two researchers independently searched three databases (PubMed, Web of Science, and Embase) in August 2020 for studies on the association between *ABCA1* 69C>T and T2DM. The following search terms were used: [(ABCA1 OR ATP-binding cassette transporter 1 OR ATP-binding cassette transporter A1 OR adenosine triphosphate-binding cassette transporter A1 OR ATP Binding Cassette Sub Family A Member 1 OR ATP Binding Cassette Transporter, Subfamily A) AND (polymorph* OR variant* OR mutation* OR genotyp* OR allele* OR SNP*) AND (diabetes mellitus OR diabet* OR NIDDM OR T2D* OR T2DM)]. The search was not restricted by publication date. Duplicates and irrelevant studies were removed through the initial screening of titles and abstracts according to the eligibility criteria. This meta-analysis was conducted according to the checklist outlined in the Preferred Reporting Items for Systematic Reviews and Meta-Analyses (19).

### Study Inclusion and Exclusion Criteria

The following criteria were used to identify eligible studies: (i) evaluating the association between the *ABCA1* polymorphisms and the risk of T2DM; (ii) using prospective or retrospective cohort study or case-control study design; (iii) providing sufficient information to calculate odds ratios (ORs) and 95% confidence intervals (CIs); and (iv) being published in English. Exclusion criteria included: (i) conference or meeting abstracts, summaries, reviews, comments, letters, news, and editorials; (ii) *in vitro* or animal studies; or (iii) unable to extract the data. In case of overlapping data, only the most recent and comprehensive data were included in the meta-analysis.

### Data Extraction and Quality Assessment

All data were extracted independently by two researchers, and discrepancies were resolved by consensus. The following information was extracted from each study: name of the first author, publication year, study design, country, the number of participants, percentage of T2DM and females, age, body mass index (BMI), genotyping method, and the Newcastle–Ottawa scale (NOS) score. Two researchers independently assessed the selected studies based on the NOS for cohort studies and case-control studies (20). NOS has three categories: selection of study sample, comparability between the case and control groups, and outcome or exposure assessment. Each study can be assessed with a total score of 0-9. In this review, we rated 1 point for each item of comparability, if age and other known risk factors (such as BMI) were matched or adjusted for in the analysis.

### Statistical Analysis

Review Manager (version 5.3; The Cochrane Collaboration, Copenhagen, Denmark) was used for data review. ORs and 95% CIs were calculated using the Z test to estimate the strength of the association between the *ABCA1* 69C>T polymorphism and the risk of T2DM. A *p* value <0.05 was considered statistically significant.

The heterogeneity across studies was estimated using a chi-square test, and an I² statistic. I² >50% was considered to indicate significant heterogeneity. In the absence of any statistical evidence of heterogeneity, the fixed-effects model was used; otherwise, the random-effects model was used to calculate pooled estimates (21, 22). Subgroup analysis was performed according to ethnic groups. Both the Begg test and the Egger regression test of the funnel plot were conducted using R Studio software (version 3.6.0; R Foundation for Statistical Computing, Vienna, Austria) to identify publication bias (23, 24).

## Results

### Literature Search and Characteristics of Included Studies

**Figure 1:**
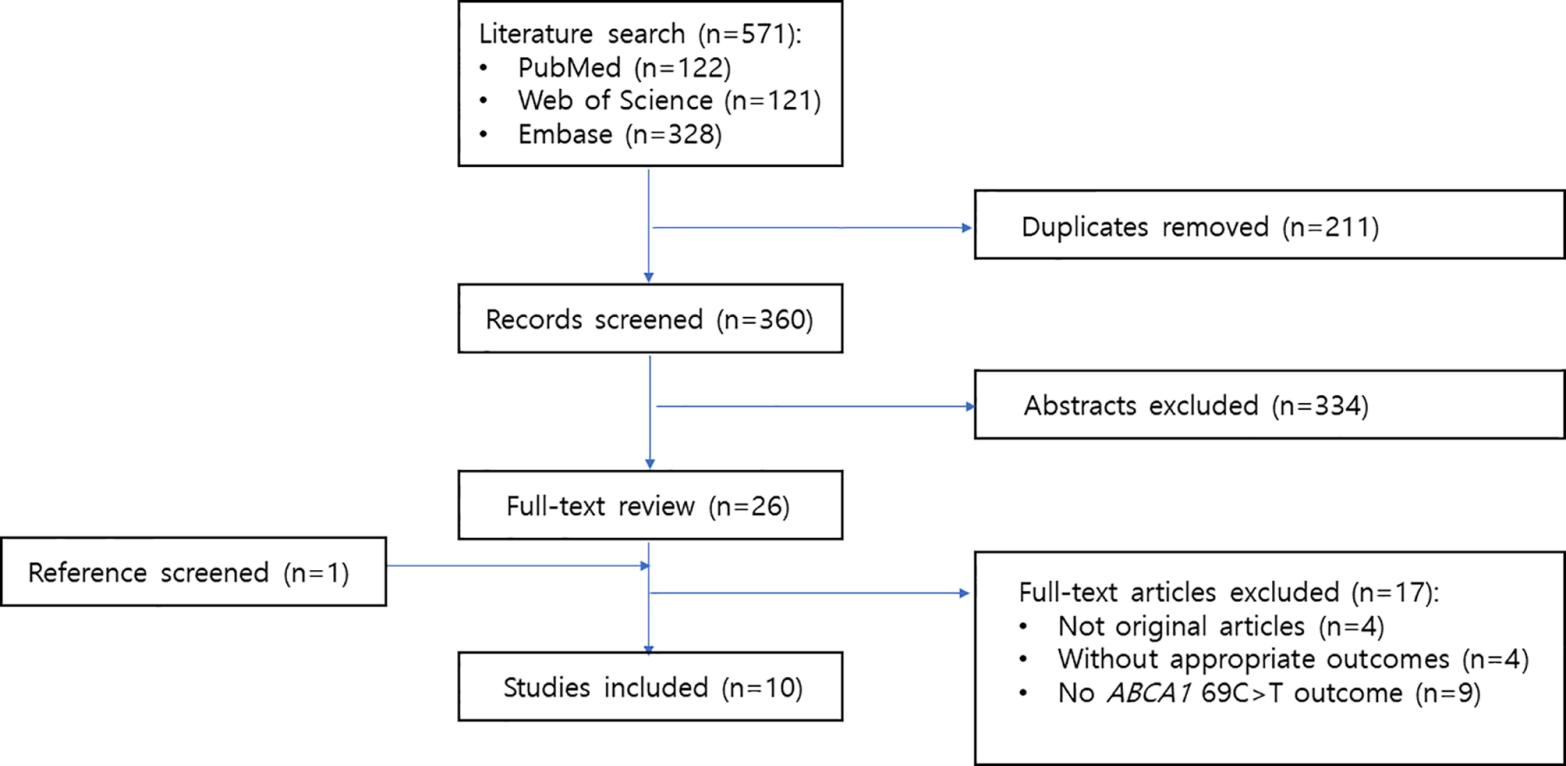
Flow diagram of included studies.

A detailed flow chart of the study selection process is presented in **Figure 1**. A total of 571 studies were retrieved through the electronic databases. After duplicate removal, 360 records were initially identified, and the titles and abstracts were screened for inclusion in the study. From this initial review, the full texts of 26 studies were assessed for eligibility. Of these studies, 17 were excluded for the following reasons: not original articles (*n* = 4), not having appropriate outcomes (*n* = 4), and not containing *ABCA1* 69C>T outcomes (*n* = 9). One study was added through manual search. Thus, 10 articles were identified for this meta-analysis. All the 10 articles were written in English.

The characteristics of the studies included are summarized in **Table 1**. The studies were published between 2012 and 2020, most of them were case-control studies, and they mainly included Asian populations. Quality scores evaluated using the NOS for all included studies ranged from 4 to 8 (**Table 1**).

**Table 1:** Characteristics of studies included in the systematic review.

First author, year, ref number	Study design	Country	Participants		Age (years) T2DM/Control(mean ± SD)	BMIT2DM/Control (kg/m^2^± SD)	Genotyping	NOS
			Total (T2DM %)	Female %T2DM/Control				
Alharbi, 2013 (25)	Case-control study	Saudi Arabia	756 (49.7)	40.2/46.8	50.6 ± 10.4/46.0 ± 7.7	29.5 ± 5.9/29.2 ± 5.5	PCR-RFLP	6
Du, 2020 (26)	Case-control study	China	1998 (49.9)	50.2/49.6	60.2 ± 8.6/59.7 ± 8.9	26.4 ± 3.2/25.1 ± 3.6	SNaPshot	7
Ergen, 2012 (27)	Case-control study	Turkey	157 (68.2)	66.4/34.0	56 (25-85)/49 (29-85)^1^	27.5 ± 5.0/25.2 ± 3.8	PCR-RFLP	6
Ghafar, 2020 (28)	Case-control study	Egypt	197 (52.8)	62.5/51.6	49.7 ± 9.0/48.0 ± 9.7	29.2 ± 4.1/22.6 ± 2.9	TaqMan real-time PCR	7
Haghvirdizadeh, 2015 (29)	Case-control study	Malaysia	329 (49.9)	37.2/47.3	62.1 ± 9.6/55.0 ± 11.8	27.9 ± 5.1/27.1 ± 6.2	PCR-HRM	5
Hasan, 2019 (30)	Case-control study	Bangladeshi	200 (51.0)	70.6/69.4	40.4 ± 1.3/39.0 ± 1.7	20.3 ± 0.4/20.9 ± 0.2	PCR-RFLP	5
Li, 2018 (31)	Case-control study	China	1122 (45.3)	40.9/42.8	55.3 ± 13.3/55.2 ± 10.2	26.1 ± 4.7/25.4 ± 4.6	matrix-assisted laser desorption/lionization time-of-flight mass spectrometry	8
Schou, 2012 (32)	Prospective cohort study	Denmark	10185 (7.8)	45.2/56.6	64 (57-71)/58 (43-69)^2^	28.9 (26.1-32.2)/24.6 (22.3-27.5)^2^	The ABI PRISM 7900HT Sequence Detection System	7
Singh, 2015 (33)	Case-control study	India	590 (47.5)	35.7/35.5	48.5 ± 14.5/49.0 ± 16.2	27.2 ± 3.3 (F),25.1 ± 4.9 (M) /25.9 ± 4.1 (F), 24.8 ± 5.5 (M)	PCR-RFLP	4
Yan 2020 (18)	Case-control study	China	2208(49.2)	51.7/49.1	58.8 ± 9.7/59.2 ± 9.9	26.8 ± 3.5/25.9 ± 3.8	SNaPshot	7

SD, standard deviation; BMI, body mass index; NOS, Newcastle–Ottawa scale; F, female; M, male; PCR, polymerase chain reaction; RFLP, restriction fragment length polymorphism; HRM, high resolution melting.

^1^ median (minimum-maximum).

^2^ median (interquartile range).

### Associations of the *ABCA1* 69C>T With T2DM

**Figure 2:**
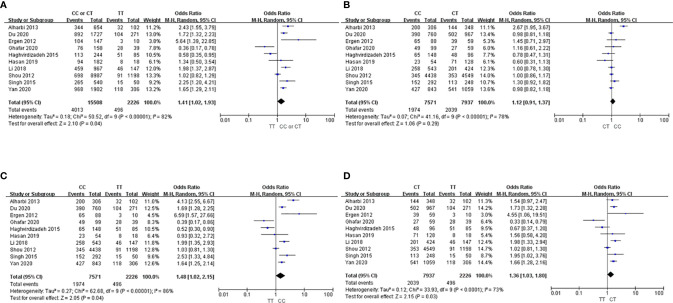
Forest plots showing the association between the *ABCA1* 69C>T genotype and T2DM risk. **(A)** CC or CT *vs.* TT, **(B)** CC *vs.* CT, **(C)** CC *vs.* TT, **(D)** CT *vs.* TT.

Ten studies with a total of 17,742 participants were evaluated for the association between *ABCA1* variants and the risk of T2DM (18, 25–33) (**Figure 2**). Because significant heterogeneity by the chi-square and I² tests (I² > 50%) was found, the analysis was conducted using the random-effects model to calculate the pooled ORs. The CC or CT genotype was associated with increased risk of T2DM than the TT genotype (OR, 1.41; 95% CI, 1.02-1.93; **Figure 2A**). Neither the Begg test nor the Egger test showed significant publication bias (Begg test, *p* = 0.655; Egger test, *p* = 0.958, **Supplementary Figure 1**). Sensitivity analysis was performed by sequentially excluding each study; the estimates showed a similar trend, with ORs ranging from 1.31 to 1.56.

In respective comparisons of the three genotypes, the CC genotype was associated with 1.1-fold (95% CI, 0.91-1.37) and 1.5-fold (95% CI 1.02-2.15) higher risk of T2DM compared to the CT and TT genotypes, respectively (**Figures 2B, C**), although statistical significance was only obtained in the comparison between the CC and TT genotypes. Regarding the CT and TT genotypes, the CT genotype had 1.4-fold (95% CI, 1.03-1.80) higher risk of T2DM compared to the TT genotype (**Figure 2D**).

### Subgroup Analysis in Asians

**Figure 3:**
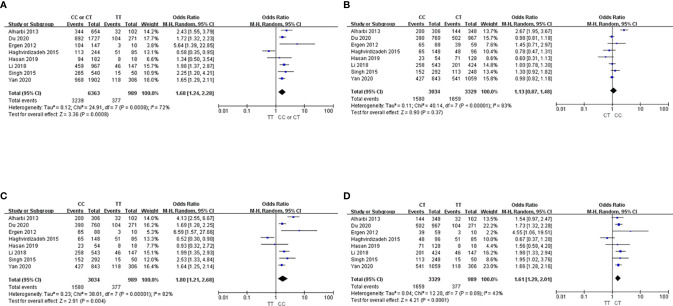
Forest plots showing the association between the *ABCA1* 69C>T genotype and T2DM risk in Asians. **(A)** CC or CT *vs.* TT, **(B)** CC *vs.* CT, **(C)** CC *vs.* TT, **(D)** CT *vs.* TT.

In the subgroup analysis for the Asian population, we found that the C allele carriers had significantly higher risk of T2DM than those with the TT genotype (OR 1.68; 95% CI, 1.24-2.28; **Figure 3A**). Although the CC genotype was associated with no significant risk of T2DM compared to the CT genotype among Asians (OR, 1.13; 95% CI, 0.87-1.48; **Figure 3B**), the CC and CT genotypes were associated with 1.8-fold (95% CI, 1.21-2.68; **Figure 3C**) and 1.6-fold (95% CI, 1.29-2.01; **Figure 3D**) higher risks than the TT genotype, respectively.

## Discussion

The inconsistency in the results regarding the association between the *ABCA1* 69C>T polymorphism and the risk of T2DM underlines the need for a meta-analysis on this topic. Therefore, we performed a meta-analysis including 10 studies and found that *ABCA1* 69TT is associated with a decreased risk of T2DM; this tendency was more pronounced in the Asian population.

ABCA1 is an efficient transporter of cholesterol from the cell to the liver and is highly expressed in β cells (17, 34). The absence of ABCA1 in β cell results in accumulation of cellular cholesterol, reduction in insulin secretion, and progressive impairment in glucose tolerance (17). In this regard, several studies investigating the association between the *ABCA1* polymorphisms and the risk of T2DM have been conducted. A meta-analysis investigating the association between *ABCA1* 219R>K polymorphism and the risk of T2DM revealed that patients with a variant allele had a lower risk of T2DM (35). Another meta-analysis on the association between the *ABCA1* 230R>C and *ABCA1* 69C>T polymorphisms and T2DM showed that these single nucleotide polymorphisms were not associated with increased susceptibility to T2DM (36). However, the aforementioned meta-analysis included data only from three studies; hence, further meta-analyses including recently published studies are warranted.

Cholesterol accumulation in β cells impairs glucose metabolism and reduces insulin secretion (16). Several clinical studies have shown that *ABCA1*C69T is associated with lipoprotein metabolism. Patients with the *ABCA1* 69CC genotype had higher plasma triacylglycerol and very-low-density lipoprotein cholesterol levels than patients with the CT genotype (37). In addition, a study that included 391 Han Chinese adults showed that patients with the *ABCA1* 69CT or TT genotype had 0.68-fold lower risk of non-alcoholic fatty liver disease than those with the CC genotype (38). In line with these studies, our results indicate that the *ABCA1* 69T allele is associated with decreased risk of T2DM.

Because eight of the 10 included studies were conducted in Asian populations, we performed a subgroup analysis for the Asian population. Similar to the overall result, the *ABCA1* 69CC or CT genotype was significantly associated with higher risk of T2DM than the TT genotype in the Asian population; the association size (OR value) was greater than that for the entire study.

Our study has some limitations. First, it was not possible to perform the subgroup analysis for the non-Asian population because only two studies were available. Second, T2DM is a complex and multifactorial disease; therefore, potential gene-gene and gene-environment interactions should be considered. However, insufficient information, including nutrition, lifestyle, and demographic details, precluded further adjustments in the analysis. Third, our meta-analysis had substantial heterogeneity, possibly because of the small number of studies included.

In conclusion, our findings indicate a significant association between the *ABCA1* 69C>T polymorphism and T2DM risk. Large-scale population-based association studies should be conducted to validate the risk indicated by our meta-analysis and investigate potential gene-gene and gene-environment interactions on T2DM risk.

## Data Availability Statement

The original contributions presented in the study are included in the article/**Supplementary Material**. Further inquiries can be directed to the corresponding author.

## Author Contributions

All the authors have made substantial contributions to the conception of the study. HY, ML, and HG contributed to designing the study. HY and ML contributed to acquisition and analysis of data. JY, GS, and HG contributed to interpretation of data. HY and ML contributed to drafting of the manuscript. HG contributed to critical revision of the manuscript. All authors contributed to the article and approved the submitted version.

## Funding

This research did not receive any specific grant from funding agencies in the public, commercial, or not-for-profit sectors.

## Conflict of Interest

The authors declare that the research was conducted in the absence of any commercial or financial relationships that could be construed as a potential conflict of interest.
