# Supplementary material for: ABCA1 69C>T Polymorphism and the Risk of Type 2 Diabetes Mellitus: A Systematic Review and Updated Meta-Analysis
Source: Front Endocrinol (Lausanne). 2021 Apr 23;12:639524. doi: 10.3389/fendo.2021.639524 (PMC8104122; doi:10.3389/fendo.2021.639524)

Supplementary figure 1. Funnel plots showing the association between the *ABCA1* 69C>T genotype and T2DM risk. (A) CC or CT vs. TT, (B) CC vs. CT, (C) CC vs. TT, (D) CT vs. TT.

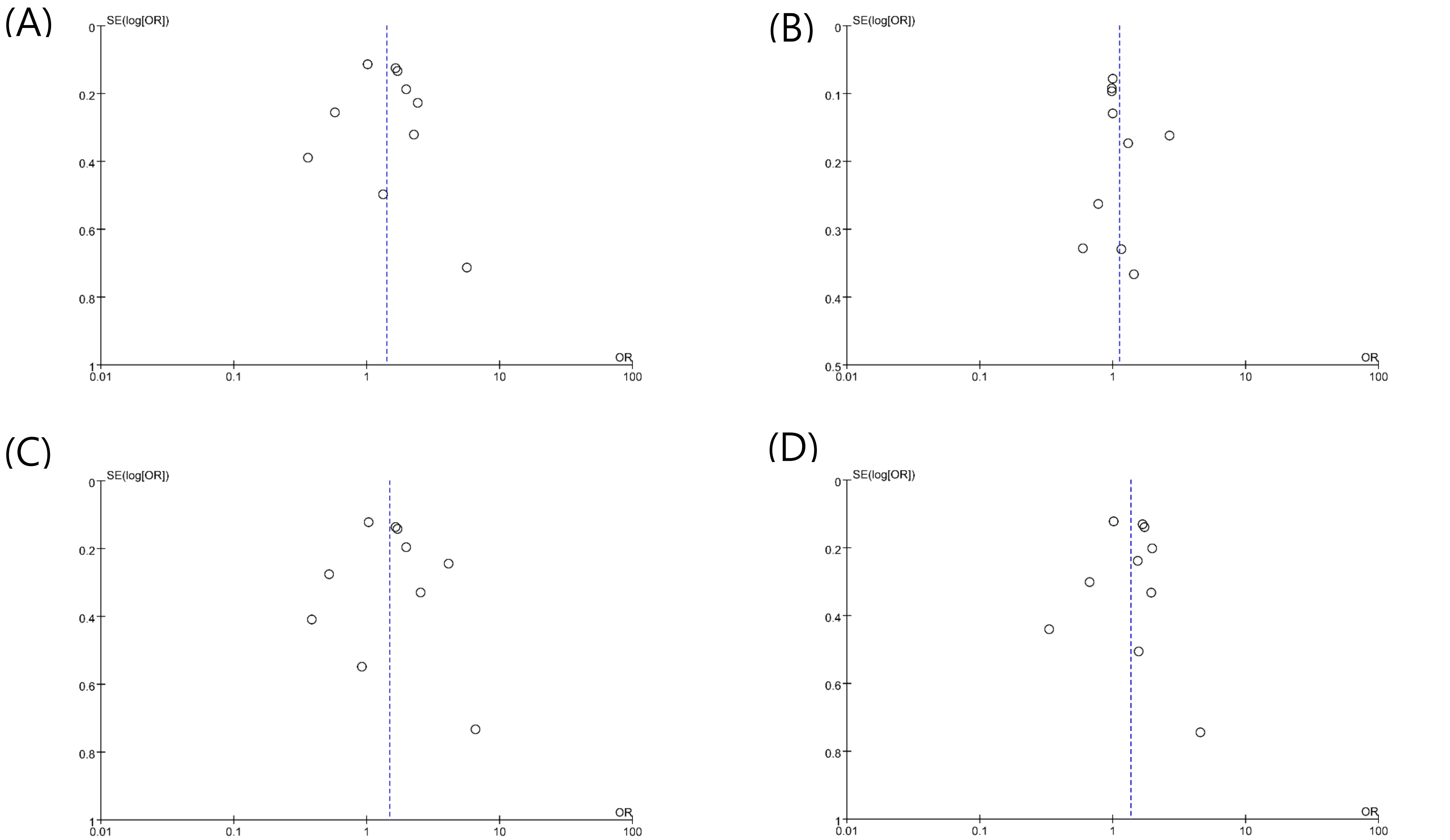

Supplement: Supplementary file 1 [file DataSheet_1.pdf]
